# Effects of enzymatic removal of plant cell wall acylation (acetylation, *p*-coumaroylation, and feruloylation) on accessibility of cellulose and xylan in natural (non-pretreated) sugar cane fractions

**DOI:** 10.1186/s13068-014-0153-3

**Published:** 2014-10-15

**Authors:** Anikó Várnai, Thales HF Costa, Craig B Faulds, Adriane MF Milagres, Matti Siika-aho, André Ferraz

**Affiliations:** Department of Chemistry, Biotechnology and Food Science, Norwegian University of Life Sciences, P.O. Box 5003, N-1432, Aas, Norway; VTT Technical Research Centre of Finland, P.O. Box 1000, Espoo, 02044 VTT Finland; Department of Food and Environmental Sciences, University of Helsinki, P.O. Box 27, Helsinki, 00014 Finland; Departamento de Biotecnologia, Escola de Engenharia de Lorena, Universidade de São Paulo, 12602-810 Lorena, SP Brasil; INRA, UMR 1163 Biodiversité et Biotechnologie Fongiques (BBF), POLYTECH, Aix Marseille Université, 163 avenue de Luminy, 13288 Marseille, Cedex 09 France; Aix-Marseille Université, INRA, BBF UMR_A 1163, 163 avenue de Luminy, 13288 Marseille, cedex 09 France

**Keywords:** Sugar cane, Acetyl xylan esterase (AXE), Feruloyl esterase (FAE), Lignin, Hydrolysis

## Abstract

**Background:**

Sugar cane internodes can be divided diagonally into four fractions, of which the two innermost ones are the least recalcitrant pith and the moderately accessible pith-rind interface. These fractions differ in enzymatic hydrolyzability due to structural differences. In general, cellulose hydrolysis in plants is hindered by its physical interaction with hemicellulose and lignin. Lignin is believed to be linked covalently to hemicellulose through hydroxycinnamic acids, forming a compact matrix around the polysaccharides. Acetyl xylan esterase and three feruloyl esterases were evaluated for their potential to fragment the lignocellulosic network in sugar cane and to indirectly increase the accessibility of cellulose.

**Results:**

The hydrolyzability of the pith and pith-rind interface fractions of a low-lignin-containing sugar cane clone (H58) was compared to that of a reference cultivar (RC). Acetyl xylan esterase enhanced the rate and overall yield of cellulose and xylan hydrolysis in all four substrates. Of the three feruloyl esterases tested, only *Ts*FaeC was capable of releasing *p*-coumaric acid, while *An*FaeA and *Nc*FaeD released ferulic acid from both the pith and interface fractions. Ferulic acid release was higher from the less recalcitrant clone (H58)/fraction (pith), whereas more *p*-coumaric acid was released from the clone (RC)**/**fraction (interface) with a higher lignin content. In addition, a compositional analysis of the four fractions revealed that *p*-coumaroyl content correlated with lignin, while feruloyl content correlated with arabinose content, suggesting different esterification patterns of these two hydroxycinnamic acids. Despite the extensive release of phenolic acids, feruloyl esterases only moderately promoted enzyme access to cellulose or xylan.

**Conclusions:**

Acetyl xylan esterase *Tr*AXE was more efficient in enhancing the overall saccharification of sugar cane, compared to the feruloyl esterases *An*FaeA, *Ts*FaeC, and *Nc*FaeD. The hydroxycinnamic acid composition of sugar cane fractions and the hydrolysis data together suggest that feruloyl groups are more likely to decorate xylan, while *p*-coumaroyl groups are rather linked to lignin. The three different feruloyl esterases had distinct product profiles on non-pretreated sugar cane substrate, indicating that sugar cane pith could function as a possible natural substrate for feruloyl esterase activity measurements. Hydrolysis data suggest that *Ts*FaeC was able to release *p*-coumaroyl groups esterifying lignin.

**Electronic supplementary material:**

The online version of this article (doi:10.1186/s13068-014-0153-3) contains supplementary material, which is available to authorized users.

## Background

Sugar cane is the main source for sucrose production in countries such as Brazil, India, and China. In Brazil alone, approximately 588 million tons of sugar cane was produced, primarily for sucrose, in the 2012/2013 harvest season [1]. Part of the recovered sucrose is used to produce ethanol as biofuel, and sugar cane bagasse is burned to produce steam and electricity. More recently, however, the recovery, hydrolysis, and fermentation of cellulose from sugar cane bagasse has gained focus for producing additional ethanol without increasing the planted area [2]. However, like most lignocellulosic materials, sugar cane bagasse is recalcitrant to enzymatic hydrolysis. A recent report indicates that sugar cane recalcitrance varies significantly with internode regions and cultivar type [3]. The central part (pith and pith-rind interface) of the internodes is less recalcitrant, especially in plants with low lignin content. Although the pith and pith-rind interface together represent only 24 to 26% of the internode sugar cane dry mass, they are interesting fractions suitable for direct (without pretreatment) enzymatic hydrolysis, giving moderate to high glucose yields. These fractions include most of the naturally occurring chemical linkages present in the lignocellulose of monocots, which is not the case with severely pretreated materials [4]. Hence, natural sugar cane fractions are attractive substrates for studying the role of accessory enzymes in experimental hydrolysis cocktails.

Understanding the fundamental structure/anatomy of plant tissues is key in predicting the enzymatic hydrolyzability of plant tissues into monomeric sugars [5]. The most common herbaceous agricultural waste materials being evaluated as raw biorefinery materials, such as corn stover, wheat straw, and sugar cane bagasse, are monocots [2,4]. In monocotyledons, the cell distribution along the axial axes of the plant is different than that observed in gymnosperms and dicotyledonous angiosperms. The cross section of internodes of monocotyledons presents vascular bundles surrounded by parenchyma cells. Each vascular bundle contains a small phloem (sieve elements and companion cells), whereas most of the bundle area is composed of vessels and fiber cells. The fibers are approximately 15 to 25 μm in diameter and 0.6 to 1.7 mm in length [6]. The parenchyma cells surrounding the vascular bundles have the main function of storing reserve materials, which in the case of sugar cane is composed mainly of sucrose.

Sugar cane internodes can be divided into three concentric layers based on the structure of plant tissues and the distribution of vascular bundles: pith, pith-rind interface, and rind (as one moves radially outwards from the middle to the epidermis [3]; see also Additional file 1: Figure S1). The inner, pith, region is rich in parenchyma cells, whereas the rind region contains more vascular bundles. Whereas parenchyma cells have bigger lumens and thinner cell walls, vascular bundles are composed of tightly packed vessel elements surrounded by sclerenchyma cells with a small lumen and thick, lignified cell walls. Apart from the chemical composition, the structure of these tissues has been shown to play a key role in the impedance of hydrolysis with enzymes. The resistance of these sugar cane fractions to hydrolysis with glycoside hydrolases has been shown to correlate with the relative area occupied by vascular bundles [3]. Consequently, the enzymatic hydrolyzability dramatically drops when moving outwards from pith to rind.

Even though the composition of the cell wall may differ by plant cell type, cellulose is embedded in a hemicellulose-lignin matrix. In annual plants, lignin presents some covalent linkages with arabinoxylan through phenolic acids, tightening the three-dimensional structure around cellulose [7–9]. Xylan may be acetylated or arabinosylated at the positions C-2 and/or C-3; the arabinose substituents are occasionally esterified with phenolic acids at the C-5 position. Phenolic acids, especially ferulic acid, aptly form direct ester-ether linkages between carbohydrates and lignin in plants, attaching lignin to the carbohydrate fraction and hence potentially forming a physical barrier to glycoside hydrolases through both substitution and steric hindrance. By fragmenting the hemicellulose-lignin network, accessory enzymes such as endoxylanase (EC 3.2.1.8), arabinosidase (EC 3.2.1.55), acetyl xylan esterase (AXE, EC 3.1.1.72), and feruloyl esterase (FAE, EC 3.1.1.73) have the potential to indirectly increase the hydrolyzability of cellulose [10,11]. Endoxylanases belonging to the glycoside hydrolase family 10 have been suggested to have lower substrate specificity and hence to be more efficient in solubilizing xylan from pretreated wheat straw than family 11 endoxylanases [12–14]. Feruloyl esterases can be classified into four types based on sequence homology and substrate specificity depending on which synthetic methyl esters of various hydroxycinnamic acids they are able to cleave and on whether they are able to release diferulic acid [15].

In this work, two sugar cane plant clones with varying composition were selected, and the two innermost fractions of their internodes (pith and pith-rind interface) were subjected to hydrolysis with mixtures of individual purified enzymes. In particular, xylanases (endoxylanase *Tm*Xyn10A from *Thermotoga maritima* and β-xylosidase *Bp*Xyn43 from *Bacillus pumilus*), acetyl xylan esterase (*Tr*AXE from *Trichoderma reesei*), and three feruloyl esterases (*An*FaeA from *Aspergillus niger*, *Ts*FaeC from *Talaromyces stipitatus*, and *Nc*FaeD from *Neurospora crassa*) were combined with a basic cellulase mixture. The hydrolytic efficiency of cellulases with and without accessory enzymes was compared.

## Results and discussion

### Chemical composition

Sugar cane clones of varying chemical composition have been grown in Lorena (SP, Brazil), characterized, and evaluated for their overall chemical composition as previously described [16]. From those clones, a hybrid with significantly lower lignin and significantly higher hemicellulose content (H58, containing 18.6 ± 0.1% of DW lignin and 26.3 ± 0.1% of DW hemicellulose) was chosen, and its susceptibility to hydrolysis was compared to that of a reference cultivar (RC, containing 24.5 ± 0.5% of DW lignin and 25.2 ± 0.4% of DW hemicellulose). These two clones were fractionated radially into four parts, and the chemical composition of these fractions was determined as described by Masarin *et al.* [16]; the composition of H58 has been previously reported by Costa *et al.* [3] (Table 1).

**Table 1:** **Chemical composition of sugar cane clones hybrid 58 (H58) and reference cultivar (RC)**

**Clone**		**H58**		**RC**	
**Component (% of DW)**		**Pith**	**Interface**	**Pith**	**Interface**
**Glucose**		53.0 ± 0.9	46.9 ± 0.7	49.2 ± 0.6	42.6 ± 0.1
**Xylose**		16.2 ± 0.4	19.9 ± 0.3	15.3 ± 0.2	18.2 ± 0.2
**Arabinose**		1.5 ± 0.1 (10.5)*	1.5 ± 0.1 (13.3)*	1.5 ± 0.0 (10.2)*	1.3 ± 0.1 (13.7)*
**Acetyl groups**		2.8 ± 0.1 (1.9)**	3.9 ± 0.1 (1.7)**	2.4 ± 0.2 (2.0)**	3.1 ± 0.0 (1.9)**
**Lignin**		12.6 ± 0.3	15.5 ± 0.3	17.2 ± 0.2	22.1 ± 0.3
**Feruloyl groups**	*ester*	0.34 ± 0.05	0.34 ± 0.05	0.47 ± 0.05	0.49 ± 0.08
	*ester and ether*	0.91 ± 0.08	0.86 ± 0.17	1.41 ± 0.12	1.55 ± 0.11
***p*** **-Coumaroyl groups**	*ester*	0.84 ± 0.09	1.02 ± 0.22	1.39 ± 0.17	1.93 ± 0.43
	*ester and ether*	3.29 ± 0.22	3.65 ± 0.39	5.06 ± 0.67	7.83 ± 0.73

*The molar ratio of xylose and arabinose units is reported in brackets.

**The molar ratio of xylose units and acetyl groups is reported in brackets.

The composition is presented as percentage of total dry weight (DW) of substrate. The sugars are given as anhydro sugars. Phenolic acid esters were measured after mild alkali extraction; the sum of phenolic acid esters and ethers was measured after severe alkali extraction [16]. The composition of the H58 fractions was adapted from Costa *et al.* [3].

The lignin content in the internal (pith and interface) fractions of the hybrid 58 (H58) sugar cane was lower, 12.6 and 15.5% of DW, than that detected in the reference cultivar (RC), 17.2 and 22.1% of DW, respectively, for the two fractions (Table 1). At the same time, H58 was enriched in both cellulose and arabinoxylan. The arabinosylation and acetylation patterns of xylan seem similar in the two clones; specifically, one arabinose unit per 10 to 11 xylose units on average in the pith and one arabinose unit per 13 to 14 xylose units in the interface region. Interestingly, acetylation of xylan seemed independent of tissue structure; the degree of acetylation was 0.4 to 0.5 in both the pith and interface regions. Due to differences in properties of the cell wall types, the lignin content of the pith region falls below 80% of that of the pith-rind interface. In general, pith, mostly composed of large parenchyma cells and thin-walled fibers, contains less lignin and more cellulose than the pith-rind interface, where parenchyma cells are smaller and thick-walled fiber elements are more abundant [6], as also reported by Costa *et al.* [3].

In general, the lower lignin content in H58 concurs with a lower extent of substitution of plant cell wall polymers with phenolic acids: overall, the low-lignin-containing hybrid H58 contained 35 to 50% less phenolic acids than the RC, despite their similar xylan content (Table 1). Of the phenolic acids, no significant difference could be observed in the distribution of ferulic acid across the internode. Similarly to other sugar cane clones studied by Costa *et al.* [3], both clones contained similar amounts of ferulic acid, coinciding with the arabinose content, in the pith and interface fractions (Table 1). On the other hand, the amount of *p*-coumaroyl groups was found to correlate with the lignin content, being lower in the pith and higher in the interface fractions of both clones. In agreement with previous observations on sugar cane [3,17], we found that *p*-coumaroyl groups were more abundant in all four sugar cane fractions (both clones) than feruloyl groups. Similar to maize, in sugar cane *p*-coumaroyl groups predominantly esterify lignin, while feruloyl groups esterify hemicellulose [17,18]. The incorporation of *p*-coumaric acid into lignin and that of ferulic acid into arabinoxylan have been described prior to polymerization of the corresponding plant cell wall polymers in maize [18,19]. Our results corroborate these observations: *p*-coumaroyl groups were more likely to be found in lignin, while feruloyl groups rather esterified xylan and linked xylan to lignin (Table 1).

### Effect of xylanase and β-xylosidase on cellulose hydrolysis in two sugar cane clones with different lignin content

The internal, pith and pith-rind interface, fractions of two sugar cane clones were hydrolyzed with mixtures of purified cellulases and of cellulases and xylanases. The cellulase mixture (C) was composed of purified cellulases *Tr*Cel7A, *Tr*Cel6A, *Tr*Cel5A, and *Tr*Cel7B in a ratio 60:20:10:10 at a total protein dosage of 5 mg/g dry weight (DW) supplemented with β-glucosidase *An*Cel3A at 10.0 IU/g DW; the xylanase mixture (X) was composed of xylanase *Tm*Xyn10A at 0.25 mg/g DW and β-xylosidase *Bp*Xyn43 at 10 IU/g DW dosage.

**Figure 1:**
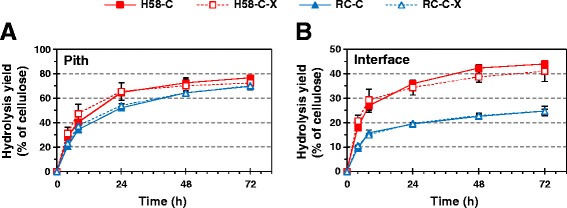
**The effect of xylanase supplementation on enzymatic glucose release from sugar cane fractions.** Hydrolysis of pith **(A)** and interface **(B)** fractions of the sugar cane clones H58 (red square marks) and RC (blue triangle marks) with cellulase mixture with (C-X; open symbols) and without (C; full symbols) xylanase. The loading of cellulases was 5 mg/g DW, of xylanase 0.25 mg/g DW. For further details see Materials and methods section.

Due to the abundance of parenchyma cells with thinner cell walls and the lower occurrence of vascular bundles, the pith was more accessible than the outer pith-rind interface fraction in both clones. The pith fraction yielded twice as much glucose with both C and C-X mixtures (70 to 77% of total glucan) than the pith-rind interface (25 to 44% of total glucan) (Figure 1), as also observed in earlier work using commercial cellulases by Costa *et al.* [3]. Due to the 27 to 30% lower lignin content in H58 (Table 1), both pith and interface fractions were hydrolyzed more readily when compared to those in the RC. An almost twofold difference in the efficiency of cellulose hydrolysis could be observed during the course of hydrolysis in the interface fractions: 25% of total glucan was released after 72 h in the RC and 41 to 44% of total glucan in H58 (Figure 1B). This difference was much less pronounced, although noticeable, between the less resistant pith fractions. After 72 h of reaction, the glucose release from the RC and H58 piths leveled at similar values: 70% of total glucan in RC and slightly higher, 73 to 77% of total glucan, in H58 with the C and C-X mixtures (Figure 1A).

Despite the high xylan content of RC, (18.2% of DW in interface and 15.3% in pith), the addition of xylanase had only a slight effect on the overall glucose release from the RC fractions after 24 h (Figure 1). As a result of xylanase addition, alteration from the yield obtained with the mixture C could only be observed in the initial phase of hydrolysis. The slight increase was, however, significant only in the initial hydrolysis (*t*-test: *P* < 0.01 at 4 h and *P* < 0.10 for RC pith at 8 h; *P* < 0.01 up to 8 h for H58 pith) of pith in RC (Figure 1A). The variation between batches of H58 internodes resulted in a high standard deviation in glucose release (Figure 1), overshadowing differences between the enzyme mixtures in conversion yield (*t*-test: *P* = 0.163 at 4 h and *P* = 0.096 at 8 h for H58 interface).

**Figure 2:**
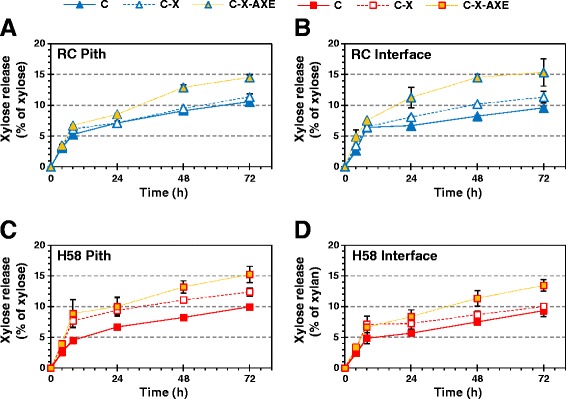
**The effect of xylanase supplementation on enzymatic xylose release from sugar cane fractions.** Xylose release from pith **(A, C)** and interface **(B, D)** fractions of RC (**A**, **B**, blue marks) and H58 (**C**, **D**, red marks) with C (solid line), C-X (dashed line), and C-X-AXE (dotted orange line) enzyme mixtures.

The fact that no significant increase in glucose release could be seen by supplementing xylanases is likely due to the promiscuity of *Tr*Cel7B, which exhibits activity on xylan [20,21]. Indeed, a release of monomeric xylose units was observed also with mixture C, containing *Tr*Cel7B but no xylanases (Figure 2). These suggest that the level of *Tr*Cel7B in mixture C was high enough to depolymerize xylan satisfactorily without additional xylanase in order to increase accessibility of cellulose. Xylanase addition, on the other hand, had a clear impact on the release of xylose monomers. Supplementing mixture X (*Tm*Xyn10A xylanase and *Bp*Xyn43 β-xylosidase) to mixture C led to a significant increase (by 25 to 71%, *t*-test: *P* < 0.01) in xylose release from the pith of the H58 clone with lower lignin content, whereas the increase was less significant (*t*-test: 0.01 < *P* < 0.03 up to 48 h) in the interface fraction of H58 (Figure 2C, D). In RC, however, the effect seemed to be the opposite: xylose release was significantly higher (by 19 to 24% after at least 24 h of incubation, *t*-test: *P* < 0.01) from the interface with the mixture C-X than C, while only a minor positive effect in the pith fraction could be detected (*t*-test: *P* > 0.10) (Figure 2A, B). The data indicate that there may be differences in the distribution and/or structure of xylan between the two clones.

### Effect of acetyl xylan esterase on hydrolysis of xylan and cellulose

**Figure 3:**
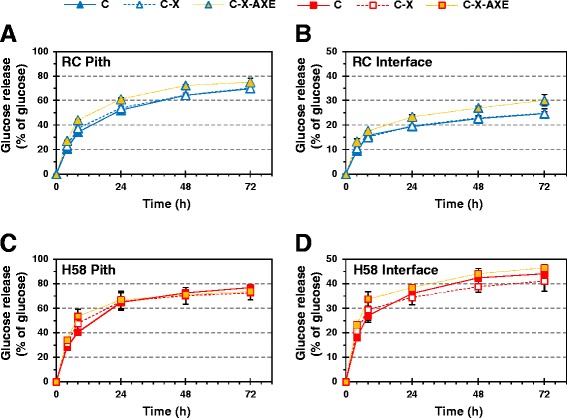
**The effect of acetyl xylan esterase supplementation on enzymatic glucose release from sugar cane fractions.** Glucose release from pith **(A, C)** and interface **(B, D)** fractions of RC (**A**, **B**, blue marks) and H58 (**C**, **D**, red marks) with C (solid line), C-X (dashed line), and C-X-AXE (dotted orange line) enzyme mixtures.

Acetyl xylan esterase has been found to enhance complete hydrolysis of acetylated xylans to xylose monomers by removing substitutions, which prevent the xylan backbone from sitting in the catalytic site of endoxylanase and β-xylosidase [22–24]. In fact, acetyl xylan esterase improves the release of not only xylose but also glucose [11] as the concomitant hydrolysis of intertwined xylan and cellulose enhances overall hydrolyzability of both polysaccharides [21,25] and as monomerization of xylooligosaccharides alleviates inhibition of cellulases by xylooligosaccharides [26]. In accordance with these features, we observed a positive impact on the release of xylose and glucose monomers during the hydrolysis of pith and interface fractions of sugar cane when the cellulase-xylanase (C-X) mixture was complemented with 0.25 mg/g DW *Tr*AXE (C-X-AXE). What is striking from the results is that *Tr*AXE improved not only the final degree (after 72 h) but also the initial rate (that is, the degree after 4 h) of hydrolysis of both xylan and cellulose in RC (Figures 2 and 3). Supplying *Tr*AXE to the C-X mixture increased cellulose conversion by 17% and 25% after 4 h and by 7% and 22% after 72 h in RC pith and interface, respectively. Moreover, the xylose release increased by 8% and 39% after 4 h and by 28% and 36% after 72 h in the corresponding fractions. Again, differences in glucose release from the clone H58 were suppressed by the large variation between batches of H58 internodes (Figure 3C-D). Reaching a higher yield at the end of hydrolysis infers that supplementing with *Tr*AXE facilitated the hydrolysis of previously inaccessible cellulose and xylan structures. At the same time glucose and xylose units were released from the substrate at a higher pace in the presence of *Tr*AXE, resulting in a higher yield of glucose and xylose even in the beginning of hydrolysis.

Despite morphological differences between the two sugar cane fractions and the clearly higher accessibility of cellulose in pith than in interface (see the twofold yield in Figure 1), the overall xylose release was fairly similar from the pith and interface fractions in both clones (Figure 2). After 72 h approximately 9 to 12% of the total xylose could be released as monomers with the C or C-X, and 14 to 15% of the total xylose with the C-X-AXE mixture. The formation of xylose monomers was significantly enhanced by *Tr*AXE for both clones (*t*-test: *P* < 0.03 after 72 h for all four fractions). In the RC fractions the effect of *Tr*AXE on xylose release seemed to be larger compared to the effect of X (Figure 2A-B). This implies that the xylooligosaccharides released by *Tr*Cel7B and *Tm*Xyn10A C-X were acetylated in a way that prevented their cleavage to monomers by β-xylosidase [22]. In agreement with the similar degree of acetylation in the pith or interface fraction of the two clones (Table 1), the pith of both clones gave similar yields with the C and C-X-AXE mixtures, although the C-X mixture seemed slightly more efficient in the pith of H58. Although cellulose was clearly more accessible in the interface fraction of the low-lignin-containing sugar cane clone (H58) than in that of RC (see the twofold yield in Figure 3B, D), none of the three enzyme mixtures (C, C-X, and C-X-AXE) could reach higher xylose release from the interface of H58 than from that of RC (Figure 2), presumably due to arabinosylation of xylan, making it more resistant to monomerization.

Similar to a previous report by Selig *et al.* [11], addition of AXE led to an increase not only in the xylose but in the glucose release in RC (Figure 2A,B and Figure 3A, B). This infers better fragmentation of xylan in the presence of *Tr*AXE, creating new cleavage sites for endoxylanase [22] and that solubilization of additional xylan facilitated concomitant hydrolysis of previously inaccessible cellulose [21]. Although the xylose release showed a similar trend in both clones, the effect of AXE on the glucose yield was significant only in RC and was less pronounced in H58 (in fact, it was significant only in the initial phase of hydrolysis due to the large variation in the H58 samples). Despite the increase in xylose release when supplementing with the AXE, no additional cellulose seems to have been solubilized, implying that deacetylation of xylan in H58 pith did not lead to the release of previously unhydrolyzed xylan and consequently cellulose structures.

**Figure 4:**
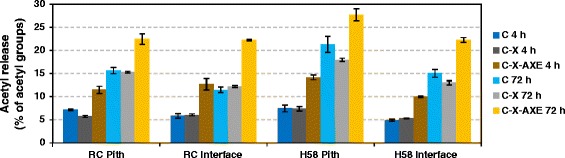
**Release of acetic acid from sugar cane with the C, C-X, and C-X-AXE enzyme mixtures.**

As a result of supplementing the C-X mixture with *Tr*AXE, the release of acetic acid was already detected in the beginning of hydrolysis (after 4 h), demonstrating the activity of AXE on sugar cane (Figure 4). Interestingly, acetic acid was also released from sugar cane when it was incubating with the C and C-X mixtures (in the absence of the AXE), and the amount gradually increased during the course of hydrolysis. By 72 h of hydrolysis, 22 to 28% of the total acetyl groups were released by C-X-AXE from all four fractions, accounting for 2.6 to 4.0 mM acetic acid in the reaction mixtures. The increase in acidity was minimal during reaction as the release of acidic groups was compensated by a high buffer capacity (50 mM sodium citrate buffer). In all four fractions the increase in release of acetic acid coincided with an increase in both glucose and xylose release (Figures 2, 3, and 4), which is in agreement with previous findings [11,22]. After 72 h of incubation with the mixture C-X-AXE, deacetylation of sugar cane occurred in general to a higher extent in the pith fractions than in the interface of the tissues (Figure 4).

### Effect of feruloyl and *p*-coumaroyl esterases on phenolic acid, xylose, and glucose release during hydrolysis of sugar cane

**Figure 5:**
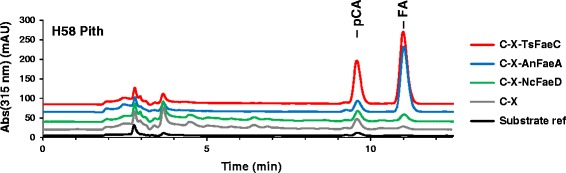
**Substrate specificity of feruloyl esterases.** The HPLC chromatograms of 72-h hydrolysates of C-X (gray line) and C-X-FAE mixtures (blue, red, and green lines for *An*FaeA, *Ts*FaeC, and *Nc*FaeD, respectively) show the separation of *p*-coumaric acid and ferulic acid released during hydrolysis of H58 pith fraction. *p*-Coumaric acid (pCA) eluted at 9.6 min; ferulic acid (FA) eluted at 11.0 min.

Feruloyl esterases have been found to increase the hydrolysis of xylan and cellulose in several crops [11,27]. We selected three feruloyl esterases belonging to different substrate specificity groups, *An*FaeA from *Aspergillus niger*, *Ts*FaeC from *Talaromyces stipitatus*, and *Nc*FaeD from *Neurospora crassa*, to compare their potential to enhance total solubilization of sugar cane. Feruloyl esterases were supplemented to the C-X mixture at a loading of 0.25 mg/g DW substrate. The three feruloyl esterases exhibited distinct substrate profiles on non-pretreated milled sugar cane. Type A and C, and to a limited extent also type D, feruloyl esterases released ferulic acid (FA) from their esters in sugar cane. The difference in ferulic acid release between these enzymes was in agreement with their specific activity against methyl ferulate, an artificial substrate; the activity of the *Nc*FaeD preparation was three orders of magnitude lower than that of *An*FaeA and *Ts*FaeC against methyl ferulate. On the other hand, in contrast to the earlier reported substrate specificities summarized by Crepin *et al.* [15], only the type C feruloyl esterase was able to release *p*-coumaric acid (pCA) from the natural substrate (Figure 5 and Table 2). The low activity of *Nc*FaeD could indicate a different substrate preference from that of *An*FaeA, active on feruloyl esters, and *Ts*FaeC, capable of hydrolyzing both feruloyl and *p*-coumaroyl esters. Our HPLC method was unsuitable for the detection and separation of phenolic acid dimers; thus, we could not confirm if phenolic acids were released only from the ester form or also from dimeric forms of the phenolic acid. In addition, with the currently available methods we could only detect and quantify the release of those hydroxycinnamic acids that are linked to biomass through a single ester linkage: hydrolysis of hydroxycinnamoyl esters also etherifying lignin will be undetected, as they remain linked to lignin.

**Table 2:** **Phenolic acids released from sugar cane after 72 h hydrolysis**

**Fraction**	**Enzyme mix**	**RC**		**H58**	
		**pCA**	**FA**	**pCA**	**FA**
		**% of pCA ester**	**% of FA ester**	**% of pCA ester**	**% of FA ester**
**Pith**	*Background (substrate only)*	*0.1*	*<0.1*	*<0.1*	*<0.1*
	C-X	1.5 ± 0.0	1.0 ± 0.3	1.0 ± 0.3	1.5 ± 1.1
	C-X-AnFaeA	1.4 ± 0.3	**18.3 ± 0.8**	1.0 ± 0.0	**46.8 ± 11.9**
	C-X-TsFaeC	**6.7 ± 0.4**	**20.9 ± 0.7**	**5.5 ± 0.5**	**48.6 ± 9.9**
	C-X-NcFaeD	1.5 ± 0.1	2.2 ± 0.5	1.1 ± 0.2	7.0 ± 4.8
**Interface**	*Background (substrate only)*	*0.3*	*<0.1*	*0.3*	*<0.1*
	C-X	1.9 ± 0.2	0.1 ± 0.2	1.6 ± 0.3	1.7 ± 0.7
	C-X-AnFaeA	1.6 ± 0.1	**9.7 ± 0.4**	1.8 ± 0.2	**34.3 ± 4.2**
	C-X-TsFaeC	**8.8 ± 0.1**	**13.4 ± 0.1**	**8.7 ± 0.4**	**34.0 ± 3.6**
	C-X-NcFaeD	2.0 ± 0.1	**1.9 ± 0.2**	1.7 ± 0.1	**4.1 ± 0.5**

The release of phenolic acids (ferulic acid, FA, and *p*-coumaric acid, pCA) is expressed as percentage of phenolic acids in ester form, as the hydrolysis conditions are too mild to cleave ether linkages. The values significantly different from the amount released by the C-X mixture are marked in bold (*t*-test; *P* < 0.01).

When combined with cellulases and xylanases at pH 5, *An*FaeA and *Ts*FaeC were equally efficient on all sugar cane fractions at releasing ferulic acid (Table 2). *An*FaeA and *Ts*FaeC released 18.3% and 20.9% of FA esters from the pith and 9.7% and 13.4% of FA esters from the interface fraction of RC, respectively. Pith was more accessible than interface not only to cellulases (Figure 1) but also to feruloyl esterases (Table 2), despite the similar ferulic acid content of these two fractions. This trend did not change between the two evaluated plants: FA release was higher from the pith (46.8% and 48.6% of FA esters) than from the interface (34.3% and 34.0% of FA esters with *An*FaeA and *Ts*FaeC, respectively) of H58 with reduced lignin content after a 72-h hydrolysis with C-X-FAE mixtures (Table 2). In fact, the extent of FA release was three times higher in each fraction of the low-lignin-containing hybrid clone H58 than in the corresponding fractions of the RC. *Nc*FaeD, although yielding approximately tenfold less free FA, followed the same pattern as the two other feruloyl esterases studied. With about 50% more FA released from the pith than from the interface in both clones, and a twofold higher FA release from the H58 compared to the RC, this could be related partly to a lower lignin content and partly to a higher extent of cellulose hydrolysis in the respective fractions, which facilitates better enzyme access to feruloylated xylan. The size of the feruloyl esterases (*An*FaeA 28.3 kDa [28], *Ts*FaeC 55.3 kDa [29], and *Nc*FaeD: 28.6 kDa [30]) did not seem to affect the hydroxycinnamic acid release, implying that the enzymes were not differentially slowed down by diffusion through the cell wall in the milled sugar cane fractions.

Of the three feruloyl esterases compared, *Ts*FaeC was the only esterase capable of solubilizing pCA from the sugar cane samples. The release of pCA was in contrast to that of FA: more pCA was solubilized from the more recalcitrant interface (8.8% and 8.7% of pCA esters) than from the pith fractions (6.7% and 5.5% of pCA esters from RC and H58, respectively); however, the pCA yields remained low (Table 2). In addition, despite the twofold glucose yield from the interface of H58 compared to that of RC, no difference in the pCA release could be observed between the two clones (Figure 1 and Table 2). As compared to the hydrolysis of feruloyl esters, that of *p*-coumaroyl esters was hindered, and the accessibility of *p*-coumaroyl esters seemed independent of cellulose hydrolysis. This suggests that *p*-coumaric acid might have been released from esters formed with lignin rather than xylan. In fact, approximately 20% more *p*-coumaroyl esters were hydrolyzed from the pith of RC with higher lignin content (17.2% of DW) than from that of the low-lignin-containing clone H58 (12.6% of DW). Recently, *Ts*FaeC has been shown to hydrolyze acetyl groups of milled wood lignin, supporting the promiscuity of *Ts*FaeC [31].

**Figure 6:**
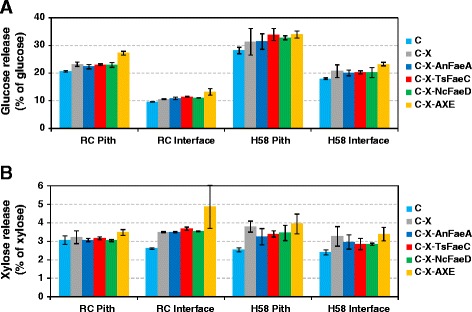
**The effect of FAE supplementation on the hydrolysis yield of glucose and xylose from sugar cane.** Glucose **(A)** and xylose **(B)** release from the pith and interface fractions of RC and H58 sugar cane clones after 4-h hydrolysis with the enzyme mixtures C, C-X, C-X-AnFaeA, C-X-TsFaeC, C-X-NcFaeD, and C-X-AXE.

Lignin is considered as one of the major factors hindering hydrolysis of lignocellulosic materials. Being attached to the carbohydrate fraction through esterifying hydroxycinnamic acid residues such as ferulic acid and *p*-coumaric acid, lignin potentially forms a physical barrier to enzymes, and impedance of hydrolysis would increase along with conversion of biomass due to accumulation of lignin. Supplementing enzyme cocktails with feruloyl esterases could potentially unzip lignin off the polysaccharide matrix, which hence could become more accessible to glycoside hydrolases. However, despite the extensive release of phenolic acids from sugar cane (34.0% of FA esters and 8.7% of pCA esters from H58 interface with *Ts*FaeC), the tested feruloyl esterases had little effect on substrate accessibility in natural (non-pretreated) sugar cane fractions (Table 2 and Figure 6). Only *Ts*FaeC, releasing both FA and pCA, increased cellulose hydrolysis significantly in the RC interface (*t*-test: *P* < 0.03 up to 8 h; *P* = 0.052 at 24 h). Supplementing any of the three feruloyl esterases tested resulted in no significant increase in glucose release during hydrolysis of the three other fractions at a 10% significance level (Figure 6). In the fractions of H58, the effect of feruloyl esterases was insignificant compared to the large variation in the samples. Therefore, it seems that removal of ferulic acid substitutions on the arabinoxylan did not affect the access of the cellulases to their site of action, signifying that the substitution pattern of feruloylation is not so dense as to form a “pseudo lignin,” entrapping the cellulases. The lack of increase in cellulose degradation could also indicate that, despite phenolic acid substitution, *Tr*Cel7B and *Tm*Xyn10A were capable of fragmenting xylans that may limit access to cellulose microfibrils.

Similarly, a moderate improvement in xylose release could be observed only from the interface fraction of RC (Figure 6). It is likely that, despite the removal of hydroxycinnamoyl groups from arabinosyl groups, the arabinosylation of the xylan backbone remained to hinder monomerization of arabinoxylan to xylose. Xylose release increased significantly only by *Ts*FaeC (*t*-test: *P* < 0.03 up to 24 h; *P* < 0.10 for 48 to 72 h), which indicates that solubilization of hydroxycinnamoyl groups from xylan or xylooligosaccharides enhanced saccharification of xylan to xylose monomers. Despite the similar efficiency of *An*FaeA and *Ts*FaeC in solubilizing ferulic acid, *Ts*FaeC seemed more effective in increasing saccharification of cellulose and xylan than *An*FaeA, which also removes diferulates [15]. A plausible explanation for this could be that, unlike *An*FaeA, *Ts*FaeC can access and remove feruloyl groups that are associated to the lignin. This in turn would allow better access of α-arabinofuranosidase and then xylanase to further degrade the xylan backbone. Hydrolysis of diferulate may not sufficiently open up the cell wall structure or remove the substitutions, and thus the overall effectiveness may be compromised in the case of *An*FaeA. The solubilization of hydroxycinnamic acids seemed to be more effective from the interface as compared to the pith in RC, despite the lower yield of FA in the outer region. In fact, in contrast with the FA yield, the pCA release with *Ts*FaeC was more substantial from the interface than from the pith of the RC, suggesting the significance of removal of potentially lignin-linked *p*-coumaroyl groups for increased substrate accessibility.

## Conclusions

Hydrolyzability of the two innermost (pith and pith-rind interface) fractions of two sugar cane clones with different lignin content were studied by supplementing cellulases with xylanases and esterases. Acetyl xylan esterase *Tr*AXE enhanced accessibility and hydrolysis of cellulose and xylan by cellulases and xylanases in all four fractions. However, the effect of feruloyl esterases was less clear. The three feruloyl esterases, types A, C, and D, had distinct product profiles on non-pretreated sugar cane substrate, indicating that sugar cane could function as a possible natural substrate for FAE activity measurements. Of the three feruloyl esterases tested, only the type C *Ts*FaeC released pCA, while the type A *An*FaeA and type C *Ts*FaeC released FA from both pith and interface fractions. FA release was higher from the less recalcitrant clone (H58)/fraction (pith), whereas more pCA was released from the clone (RC)/fraction (interface) with higher lignin content. In addition, compositional analysis of the four fractions revealed that *p*-coumaroyl content correlated with lignin, while feruloyl content correlated with arabinose content, suggesting differences in the esterification patterns of these two hydroxycinnamic acids. In sugar cane, feruloyl groups may also be more likely to decorate xylan, while *p*-coumaroyl groups decorate lignin, as in other grass species. The hydrolysis data suggest that *Ts*FaeC was able to release *p*-coumaroyl groups associated with the lignin. Despite the extensive release of phenolic acids, feruloyl esterases only moderately promoted enzyme access to cellulose or xylan.

## Materials and methods

### Substrates

Two sugar cane clones, a wildly grown sugar cane cultivar (reference cultivar, RC) and a hybrid plant with reduced lignin content (hybrid 58, H58), were selected as described in Masarin *et al.* [16]. The plants used in the present work were harvested in 2011 and kept frozen prior to sample preparation. The internodes of the frozen sugar cane stalks were sliced and fractionated to outermost fraction, rind, pith-rind interface (interface for short), and pith as described earlier [3] (see fractionation also in Additional file 1: Figure S1). Sucrose was extracted with hot water using a Soxhlet extractor until the sucrose level in the eluent gave a negative color result in a phenol/sulfuric acid assay [32]. After drying at room temperature, the fractions were milled in a knife mill (mesh 20).

### Enzymes

The cellobiohydrolases *Tr*Cel7A and *Tr*Cel6A and the endoglucanases *Tr*Cel5A and *Tr*Cel7B from *Trichoderma reesei* were purified according to Suurnäkki *et al.* [33], and the β-glucosidase *An*Cel3A from *Aspergillus niger* according to Sipos *et al.* [34]. The endoxylanase *Tm*Xyn10A from *Thermotoga maritima* and β-xylosidase *Bp*Xyn43 from *Bacillus pumilus* were purchased from Megazyme International (Bray, Republic of Ireland), and the acetyl xylan esterase *Tr*AXE of carbohydrate esterase family CE5 from *Trichoderma reesei* was purified according to Sundberg and Poutanen [35]. The feruloyl esterase from *Aspergillus niger* (*An*FaeA) was expressed in yeast and freeze-dried [28]. The esterases from *Talaromyces stipitatus* (*Ts*FaeC) and from *Neurospora crassa* (*Nc*FaeD) were a kind gift from Biocatalysts Ltd (Cardiff, Wales), and were produced as previously described [29,30]. The activities of the enzyme preparations were 18 U/mg (*An*FaeA), 4.25 U/mg (*Ts*FaeC), and 0.005 U/mg (*Nc*FaeD) against methyl ferulate (3-methoxy-4-hydroxycinnamic acid). The enzymes were resuspended in 50 mM sodium citrate buffer (pH 5.0) prior to hydrolysis.

### Enzymatic hydrolysis

The internal fractions, pith and pith-rind interface, of the two sugar cane clones (RC and H58) were hydrolyzed with mixtures of purified enzymes composed of cellulases, cellulases and xylanases, or cellulases and xylanases supplemented by either acetyl xylan esterase (AXE) or one of the feruloyl esterases (*An*FaeA, *Ts*FaeC, or *Nc*FaeD). The cellulase mixture (C) was composed of purified cellulases *Tr*Cel7A, *Tr*Cel6A, *Tr*Cel5A, and *Tr*Cel7B in a ratio 60:20:10:10 at an overall protein dosage of 5 mg/g DW, which was supplemented with β-glucosidase *An*Cel3A at 10.0 IU/g DW. The cellulase-xylanase mixture (C-X) consisted of the cellulase mixture, supplemented with the endoxylanase *Tm*Xyn10A at 0.25 mg/g DW and β-xylosidase *Bp*Xyn43 at 10 IU/g DW dosage. The C-X mixture was further supplemented with 0.25 mg/g DW acetyl xylan esterase (C-X-AXE), *An*FaeA (C-X-FaeA), *Ts*FaeC (C-X-FaeC), or *Nc*FaeD (C-X-FaeD). The hydrolysis was carried out in closed 15-mL Falcon tubes in triplicates in 5 mL total volume containing 2% substrate (20 mg dry substrate per mL) suspended in 50 mM sodium citrate buffer with the pH adjusted to 5.0, in a 45°C water bath shaken at 100 rpm. Samples (500-μL aliquots each) were taken with cut pipette tips after 4, 8, 24, 48, and 72 h. For measuring the background, the substrate was incubated without enzymes for 72 h. All samples were boiled for 10 min to inactivate enzymes, and centrifuged at 7,800 g for 5 min to separate substrate residues; the supernatants were collected and kept frozen prior to further analysis.

### Analysis of composition of substrates and hydrolysis products

The composition of sugar cane fractions was analyzed as described earlier [16,36]. Substrates were subjected to 72% (w/V) sulfuric acid at 30°C for 1 h, then diluted to a final acid concentration of 3% (w/V) and autoclaved at 121°C for 1 h [36]. Sugars (cellobiose, glucose, xylose, and arabinose) and acetic acid released during acid or enzymatic hydrolysis were separated and quantified using a Waters high performance ion exclusion liquid chromatography (HPLC) system with an Aminex HPX-87H column (Bio-Rad, Hercules, CA, USA) equipped with a pre-column, Waters column heater module (equilibrated at 45°C), Waters 1515 isocratic HPLC pump, and Waters 2414 refractive index detector (temperature 35°C) with 5 mM H_2_SO_4_ eluent and 0.6 mL/min eluent flow [36]. To determine the hydroxycinnamic groups in the substrates, the pith and interface fractions were subjected to mild (hydrolyzing only ester linkages) and severe alkali extraction (hydrolyzing both ester and ether linkages of hydroxycinnamic acids) as described by Masarin *et al.* [16]. Hydroxycinnamic acids (or phenolic acids) solubilized from substrates by alkali extraction or enzymatically in hydrolysis experiments were identified and quantified using ÄKTA Purifier block (GE Healthcare Life Sciences, Piscataway, NJ, USA) high performance liquid chromatography (HPLC) with a ThermoScientific ODS Hypersil column (SN:0980915U) equipped with a P-900 pump unit and UV-900 multiple wavelength monitoring unit, using freshly prepared acetonitrile:water (1:4) eluent containing 1% (V/V) acetic acid at 0.8 mL/min flow rate [16].

## Additional file

Additional file 1: Figure S1. Preparation of sugar cane fractions from sugar cane internodes. The sugar cane internodes were peeled to remove epidermis **(A)**, then divided into three concentric layers, rind, pith-rind interface, and pith **(B)**, moving inwards from the outer epidermis layer, as described earlier by Costa *et al.* [3]. The separated fractions are shown below.

## Abbreviations

AXE: acetyl xylan esterase
DW: dry weight
FA: ferulic acid
FAE: feruloyl esterase
HPLC: high performance ion exclusion liquid chromatography
pCA: *p*-coumaric acid
RC: reference cultivar
