# Supplementary material for: Effects of enzymatic removal of plant cell wall acylation (acetylation, p-coumaroylation, and feruloylation) on accessibility of cellulose and xylan in natural (non-pretreated) sugar cane fractions
Source: Biotechnol Biofuels. 2014 Oct 15;7:153. doi: 10.1186/s13068-014-0153-3 (PMC4201721; doi:10.1186/s13068-014-0153-3)
Supplement: Additional file 1: Figure S1. — Preparation of sugar cane fractions from sugar cane internodes. The sugar cane internodes were peeled to remove epidermis (A), then divided into three concentric layers, rind, pith-rind interface, and pith (B), moving inwards from the outer epidermis layer, as described earlier by Costa et al. [3]. The separated fractions are shown below. [file 13068_2014_153_MOESM1_ESM.pdf]

Additional file 1

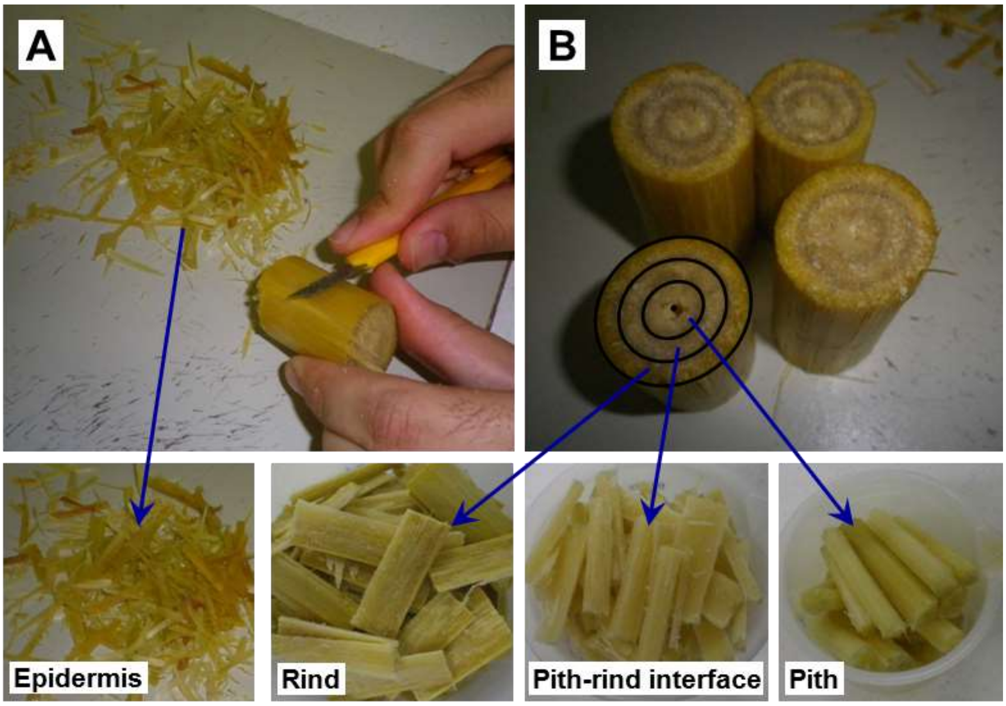

Figure S1 Preparation of sugar cane fractions from sugar cane internodes. The sugar cane internodes were peeled to remove epidermis **(A)**, then divided into three concentric layers, rind, pith-rind interface, and pith **(B)**, moving inwards from the outer epidermis layer, as described earlier by Costa *et al.* [3]. The separated fractions are shown below.
